# Advances in respiratory physiology in mouse models of experimental asthma

**DOI:** 10.3389/fphys.2023.1099719

**Published:** 2023-03-16

**Authors:** Olivia R. Carroll, Amber L. Pillar, Alexandra C. Brown, Min Feng, Hui Chen, Chantal Donovan

**Affiliations:** ^1^ Hunter Medical Research Institute, The University of Newcastle, Newcastle, NSW, Australia; ^2^ Faculty of Science, School of Life Sciences, University of Technology Sydney, Sydney, NSW, Australia

**Keywords:** asthma1, lung function2, airway hyperresponsiveness3, mouse models4, severe asthma5

## Abstract

Recent advances in mouse models of experimental asthma coupled with vast improvements in systems that assess respiratory physiology have considerably increased the accuracy and human relevance of the outputs from these studies. In fact, these models have become important pre-clinical testing platforms with proven value and their capacity to be rapidly adapted to interrogate emerging clinical concepts, including the recent discovery of different asthma phenotypes and endotypes, has accelerated the discovery of disease-causing mechanisms and increased our understanding of asthma pathogenesis and the associated effects on lung physiology. In this review, we discuss key distinctions in respiratory physiology between asthma and severe asthma, including the magnitude of airway hyperresponsiveness and recently discovered disease drivers that underpin this phenomenon such as structural changes, airway remodeling, airway smooth muscle hypertrophy, altered airway smooth muscle calcium signaling, and inflammation. We also explore state-of-the-art mouse lung function measurement techniques that accurately recapitulate the human scenario as well as recent advances in precision cut lung slices and cell culture systems. Furthermore, we consider how these techniques have been applied to recently developed mouse models of asthma, severe asthma, and asthma-chronic obstructive pulmonary disease overlap, to examine the effects of clinically relevant exposures (including ovalbumin, house dust mite antigen in the absence or presence of cigarette smoke, cockroach allergen, pollen, and respiratory microbes) and to increase our understanding of lung physiology in these diseases and identify new therapeutic targets. Lastly, we focus on recent studies that examine the effects of diet on asthma outcomes, including high fat diet and asthma, low iron diet during pregnancy and predisposition to asthma development in offspring, and environmental exposures on asthma outcomes. We conclude our review with a discussion of new clinical concepts in asthma and severe asthma that warrant investigation and how we could utilize mouse models and advanced lung physiology measurement systems to identify factors and mechanisms with potential for therapeutic targeting.

## 1 Asthma epidemiology and pathophysiology

Asthma affects approximately 10% of the population and has higher prevalence in developed countries (To et al., 2012). The World Health Organization (WHO) reports that 455,000 deaths were attributed to asthma in 2019, and that asthma is the most common chronic disease affecting children (WHO, 2022). The global prevalence of asthma varies between sources, including the Global burden of disease, Centers for Disease Control and Prevention, the Global Initiative for Asthma, and European Respiratory Society/American Thoracic Society reports, at 224–309 million people, 7.8%, 1%–18%, and 5%–10%, respectively (Diseases and Injuries, 2020; Global Initiative for Asthma, 2021; Louis et al., 2022). Asthma in adulthood is both under- and over-diagnosed, which may be attributed to multiple definitions of the disease and suboptimal adherence to diagnostic guidelines (Aaron et al., 2018; Akinbami et al., 2020; Louis et al., 2022). Globally, around 3%–10% of asthma is classified as severe, which predominantly affects females (Busse et al., 2000; The ENFUMOSA cross, 2003; Larsson et al., 2018). A cross-sectional study reported that within a randomly selected cohort, 1.1% had severe asthma and of the asthmatic cohort, 9.5% had severe disease (Ronnebjerg et al., 2021).

The pathophysiology of asthma is underpinned by chronic airway inflammation. Genetic, epigenetic, environmental risk factors, and a combination of innate and adaptive immune cells (including macrophages, neutrophils, innate lymphoid cells, dendritic cells, helper T cells [T_H_]1, T_H_2, T_H_17 and T_H_22 and follicular helper T cells [T_FH_]) are linked with asthma pathogenesis and severity. Severe asthma is heterogeneous and can be broadly characterized by different mechanisms (endotypes; type-2 [T2] high, T2 low, or non-T2) and various clinical presentations (phenotypes) (Kuruvilla et al., 2019). These mechanisms and factors contribute to changes in histopathology, including increased airway collagen deposition, mucus cell hypersecretion metaplasia, airway smooth muscle (ASM) hypertrophy/hyperplasia, reduced lung function, and airway hyperresponsiveness (AHR), and ultimately cumulate into symptoms of chest tightness, wheezing and/or shortness of breath.

### 1.1 Cellular pathophysiology

The T2 high endotype of asthma (commonly associated with the allergic phenotype) is comprised of two phases: the early (sensitization) and late (effector phase or challenge) phases. In the early phase, alarmins are released by epithelial cells in response to allergens and can induce cytokine production in type-2 innate lymphoid cells. Dendritic cells uptake allergens, migrate to lymph nodes, and present antigenic components to naïve T cells, which induces T_H_2 and T_FH_ cell differentiation. Historically, T_H_2 cells have been recognized for their role in T2 cytokine production (interleukin [IL]-4, −5 and −13) in airway inflammation, airway eosinophil infiltration, immunoglobulin E class switching and AHR (Pope et al., 2001; Agrawal and Shao, 2010; Kudo et al., 2013; Doran et al., 2017). T_FH_ cells are also implicated in mediating the early phase of allergic asthma. However, unlike T_H_2 cells, T_FH_ cells predominantly produce IL-4 and IL-21 (Coquet et al., 2015; Kobayashi et al., 2017). T_FH_ cells also produce IL-17 and have been shown to induce IgE class switching in severe asthma (Zaretsky et al., 2017). T_H_2 and T_FH_ cells support IgE antibody production and B cell maturation. Deficiency in Bcl6, the master regulator in CD4^+^ T cells, reduces T_FH_ numbers and IgE, but does not affect T2 cytokine responses or eosinophilic inflammation in the airways (Kobayashi et al., 2017), highlighting the function of these two T helper cell subsets in driving allergic asthma. This is reinforced by evidence of T_H_2 differentiation in response to allergens in the absence of IL-4 (Jankovic et al., 2000).

The late phase of allergic asthma is an established T2 response, characterized by eosinophil hematopoiesis, chemoattraction and activation, induced by granulocyte-macrophage colony-stimulating factor, IL-3 and -5. AHR presents at this stage, alongside features of epithelial damage, tissue remodeling, and chronic airway inflammation. Epithelial to mesenchymal transition presents after recurring exacerbations, which results in airways remodeling (Kudo et al., 2013). This, in conjunction with airway narrowing and tissue inflammation, contributes to symptomatic dyspnea, wheezing or whistling upon exhalation, and chest tightness (James and Wenzel, 2007; Wenzel, 2016; Quirt et al., 2018). Tissue resident mast cell numbers increase and express Fc_ε_RI, a high affinity IgE receptor. Basophil granulocytes are coated with IgE and IgE cross-linking occurs (He et al., 2013). Pro-inflammatory responses are then mediated by leukotrienes, prostaglandins, neuropeptides (substance P and neurokinin A), calcitonin gene related peptide, and histamine (Kim et al., 2018).

The T2 low endotype of asthma (T_H_1 and T_H_17 high) is associated with more severe disease, which may progress to become steroid-insensitive. Patients with severe asthma have lower atopy than patients with non-severe asthma, and IgE, blood eosinophil numbers and fractional exhaled nitric oxide (FeNO) do not differentiate severity (Moore et al., 2007; Guan et al., 2013). Notably, blood and airway neutrophil numbers have been shown to be increased in severe asthma (Crisford et al., 2021). Severe asthma presents with lower lung function and marked bronchodilator reversibility compared to mild-moderate disease. Symptoms typically present daily and disease duration is longer with sinusitis and pneumonia (Moore et al., 2007).

Respiratory infections increase susceptibility to severe asthma development, which can become steroid-insensitive (Wark et al., 2002; Hansbro et al., 2004; Cho et al., 2005). Airway epithelial cell bacterial colonization with *Chlamydia pneumoniae*, *mycoplasma pneumoniae* and staphylococcal endotoxins have been linked with asthma severity in both allergic and non-allergic endotypes. Furthermore, enterotoxins released by *Staphylococcus aureus* may act as sensitizing agents and superantigens that can activate T and B cells in human mucosa, that have been associated with severe asthma (Bachert and Zhang, 2012; Sintobin et al., 2019). *Haemophilus influenzae* is also implicated in the pathogenesis of severe disease (Simpson et al., 2007; Wood et al., 2010; Green et al., 2014). In addition to bacterial infections, respiratory viral infections such as respiratory syncytial virus (RSV), rhinovirus and influenza*,* have also been shown to contribute to asthma severity (Wark et al., 2002; Hansbro et al., 2008; Liao et al., 2016) and it is now well established that rhinovirus and RSV are risk factors for the development of atopic and non-atopic asthma, respectively (Mikhail and Grayson, 2019). Furthermore, rhinovirus, RSV, and influenza can exacerbate established asthma (Mikhail and Grayson, 2019), which can also become steroid-insensitive. Irrespective of the endotype or phenotype of asthma, the severity of asthma can be associated with increased potency of bronchoconstrictors but also an increase in the maximum contraction of ASM that can lead to complete airway closure in fatal asthma (Woolcock et al., 1998). Therefore, improving outcomes for patients requires an understanding of the mechanisms controlling airway reactivity and the influence of inflammatory mediators.

### 1.2 Anatomical pathophysiology

Changes in lung function, encompassing AHR and baseline airflow obstruction, in asthma and severe asthma are underpinned by many disease-driving factors. AHR is underpinned by hypersensitivity and hyperreactivity of smooth muscle to various stimuli [non-specific irritants, pharmacological agents (e.g., methacholine), and inflammatory mediators (e.g., histamine, leukotrienes from mast cells)], as well as impaired bronchodilator responses. AHR can occur on the background of inflammation and/or remodeling, however, this is not always the case and highlights that AHR can occur both dependently and independently of inflammation and remodeling in people with asthma and in mouse models of experimental disease, irrespective of disease severity.

Clinically, AHR is defined as a 20% decline in forced expiratory volume in 1 s (FEV_1_) after bronchoprovocation (Lotvall et al., 1998). The paradigm of AHR pathophysiology comprises tissue remodeling and structural changes to the airways and parenchyma. AHR can occur in non-asthmatics and thus may not always be indicative of asthma, however, AHR has been correlated with asthma severity (Deykin et al., 2007). ASM hypertrophy and hyperplasia, altered ASM calcium signaling, changes in epithelial cell composition, and nervous system activation, are also contributing factors to AHR. As both asthma and severe asthma are highly heterogenous, the contribution of one or more of these disease drivers to changes in lung function is patient-specific. However, persistent AHR can often coincides with airway remodeling, thus in severe asthma, and particularly in steroid-insensitive phenotypes, more severe AHR may arise from being unresponsive to current therapies.

Chronic inflammation drives smooth muscle tone, bronchospasm, edema, and mucus secretion. Airways mucus plugging also substantially contributes to asthma-related mortality rates (Worren et al., 2018). Other asthmatic phenotypes, such as obesity-associated asthma, can result in restrictive lung physiology as breathing at low lung tidal volumes increases AHR, where actin-myosin cross-linking in ASM may contribute to resistance from muscle stiffening (Fredberg et al., 1997). Neurological and psychological factors have also been linked to asthma presentation (Rosenblat et al., 2022).

It is also important to consider the contributions of different regions of the lung to AHR, including the small airways. In asthmatic patients, the small airways have greater inflammation (as measured by increased eosinophil numbers) than the larger airways (Hamid et al., 1997) and increased baseline resistance when compared to the small airways of non-asthmatics (Wagner et al., 1998). *In vivo* exposure of these small airways in asthmatic subjects to histamine resulted in greater increases in airway resistance and less reversibility with a non-selective β_1_ and β_2_-adrenoceptor agonist when compared to responses in non-asthmatic subjects (Wagner et al., 1998). Increased airway resistance can be associated with increased inflammation (Hamid et al., 1997) and airway wall remodeling (Burgel, 2011). *In vitro* studies have also shown that the small airways are more sensitive to contractile mediators (Mechiche et al., 2003), and less sensitive to β_2_-adrenoceptor agonists, than the large airways (Finney et al., 1985). However, whether there are differences in small airways AHR across the different endotypes and/or phenotypes of asthma, remains to be determined.

### 1.3 Mechanisms of AHR

#### 1.3.1 Structural changes

The lung structural changes of patients with asthma can include epithelial cell shedding, goblet cell hypertrophy, basement membrane thickening, increased ASM size (hypertrophy) and growth (hyperplasia), and increased vascularization (Hall and Agrawal, 2014; Szefler, 2015), that are further increased in severe asthma patients (Benayoun et al., 2003; Woodruff et al., 2004; Pepe et al., 2005). This relationship precipitated the dogma that AHR in asthma is caused by increased ASM hypertrophy and hyperplasia. However, some studies have shown that reducing the amount of ASM in the lungs of patients with asthma using bronchial thermoplasty does not improve overall lung function (Castro et al., 2010). Thus, more/larger ASM is not the sole factor contributing to AHR, which raises the question of what other factors contribute to increased AHR.

#### 1.3.2 Altered ASM calcium signaling

In asthma, ASM can be hyperproliferative, hypersecretory, and/or hypercontractile. ASM can be hypercontractile due to intrinsic changes in actin-myosin cross-linking, and can also release extracellular matrix factors (such as collagen types I and III, fibronectin, and elastin), cytokines that can promote inflammation (Belvisi et al., 1997; John et al., 1998; McKay et al., 2000; Hirst, 2003), and chemokines that can recruit immune cells (e.g., eotaxin). ASM heterogeneity may also be present between large and small airways, where ASM hypertrophy in large airways occurs in both non-fatal and fatal cases of asthma, and ASM hyperplasia occurs in both large and small airways in fatal asthma only (James et al., 2012). Importantly, both of these features are associated with increases in extracellular matrix deposition (James et al., 2012). However, whether these ASM changes are asthma endo/phenotype-specific is yet to be determined.

**FIGURE 1:**
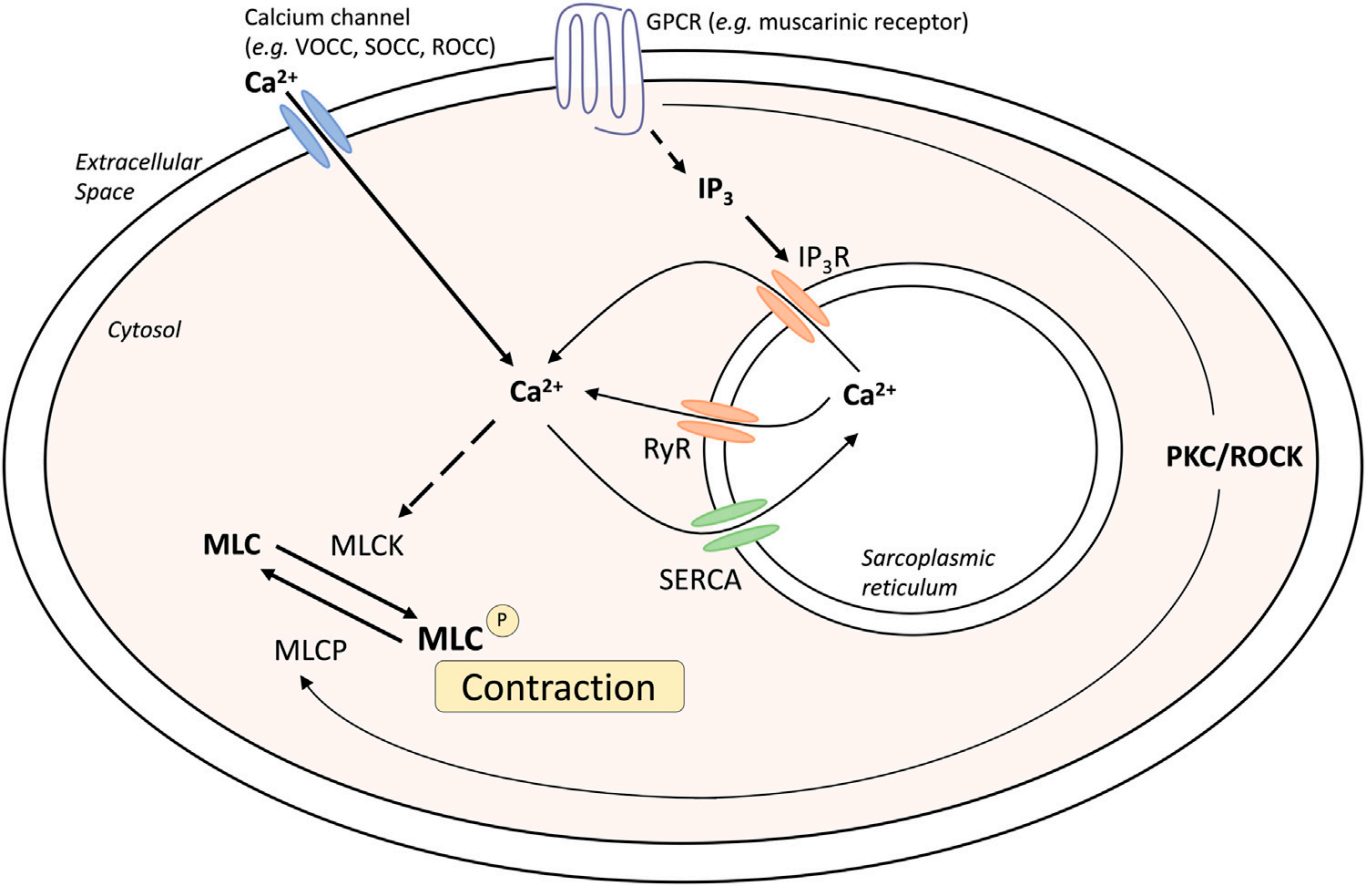
Mechanisms of airway smooth muscle contraction. Abbreviations: IP3, inositol triphosphate; GPCR, G-protein coupled receptor; MLC, myosin light chain; MLCK, myosin light chain kinase; MLCP, myosin light chain phosphatase; PKC, Protein kinase C; ROCC, receptor operated calcium channel; ROCK, Rho associated coiled-coil containing protein kinase; RyR, Ryanodine receptor; SERCA, Sarco/endoplasmic reticulum Ca(2^+^)-ATPase; SOCC, storage operated calcium channel; VOCC, voltage operated calcium channel.

ASM tone and hypercontractility can be driven by calcium signaling pathways: the calcium oscillation pathway and the ‘calcium sensitivity’ pathway. These can be activated by contractile agonists such as methacholine, and these pathways are fundamentally similar in both human and mouse airways (Bai et al., 2007; Ressmeyer et al., 2010) (Figure 1). Calcium oscillations involve calcium release and reuptake between the sarcoplasmic reticulum (SR) and the cytosol. The release of calcium from the SR into the cytosol occurs through the activation of the inositol trisphosphate receptors (IP_3_R) and/or ryanodine receptors (RyR), whereas reuptake occurs via the sarco/endoplasmic reticulum Ca^2+^–ATPase (SERCA). Increases in cytosolic calcium result in the activation of calmodulin and myosin light chain kinase, resulting in myosin light chain phosphorylation and muscle contraction. In contrast, calcium sensitivity involves signaling through protein kinase C or rho-activated kinase, that can phosphorylate and inactivate myosin light chain phosphatase (MLCP). The resulting contractile apparatus becomes more sensitive to Ca^2+^ and contraction.

Changes in intracellular Ca^2+^ levels can be detected using confocal or two-photon microscopy in ASM cells within precision cut lung slices (PCLS) loaded with fluorescent Ca^2+^ indicator dyes, such as Oregon green (Sanderson et al., 2008). This allows for assessment of contractile responses to agonists such as methacholine. Importantly, muscle contraction in response to methacholine occurs when calcium oscillations are abolished by treatment with caffeine and ryanodine to clamp intracellular calcium ([Ca^2+^]_i_) levels. Under these conditions, airway contraction is solely induced by increased Ca^2+^-sensitivity, whereby reduced MLCP activity maintains muscle tone (Bai and Sanderson, 2006).

Extracellular calcium can also contribute to ASM tone. Calcium can enter the cytosol through L-type voltage-operated Ca^2+^-channels in response to membrane K^+^ channel-dependent depolarization (Marthan et al., 1989), receptor-operated channels or ion channels, or store-operated calcium channels. Although calcium signaling has been found to be altered in disease states such as asthma and chronic obstructive pulmonary disease (COPD), this does not appear to be related to changes in receptor expression or levels, but rather increased sensitivity to the changes in calcium within the muscle.

#### 1.3.3 Role of inflammation in AHR

It is now well established that inflammation and AHR can occur independently in people with asthma or severe asthma and in murine experimental asthma and severe asthma. However, in some cases, increased presence of inflammatory cells and inflammatory cytokines/chemokines, can contribute to increased AHR. Macrophages and neutrophils can increase AHR through their release of cytokines such as IL-13. Inflammatory cytokines and chemokines can also be released from structural cells, such as ASM, within the lungs. IL-13 can directly act on the IL-13R and IL-4R on ASM to increase inflammation by up-regulating pro-inflammatory cytokines, such as IL-1β and TNFα, and directly enhancing G-protein coupled receptor agonist-associated calcium signaling (Kibe et al., 2003; Tliba et al., 2003; Vargaftig and Singer, 2003). Furthermore, IL-13 can induce AHR through epithelial changes, including increased swelling, permeability, rigidity, and mucus production (Kirstein et al., 2010). IL-13-induced AHR has also been observed in response to non-calcium altering agonists such as potassium chloride, and it has been postulated that IL-13 can alter contractile machinery in ASM by enhancing sensitivity of myofilaments or rearranging structural cytoskeleton. IL-13 can also induce AHR without increasing inflammation (Yang et al., 2001; Venkayya et al., 2002), and this is thought to be via altering gene expression of other resident airway cells, including epithelial cells and ASM cells (Laporte et al., 2001; Lee et al., 2001; Kuperman et al., 2002; Venkayya et al., 2002).

### 1.4 *In vivo* techniques for measuring AHR

Lung function measurements, including the forced oscillation technique (FOT), forced expiration (spirometry in humans, negative pressure-driven forced expiration in mice), and AHR to provocation, are commonly used as diagnostic tools in humans, and as tools to assess the severity of respiratory disease and response to treatments in pre-clinical models (predominantly in mice). FOT and forced expiration for the purposes of measuring baseline lung function has been addressed elsewhere (Devos et al., 2017; Bonnardel et al., 2019), thus, this review is focused on AHR.

In mice, AHR is commonly measured following provocation with a contractile stimulus (such as methacholine) and this is an invasive and terminal procedure. The most commonly used invasive plethysmographs to measure airway resistance to methacholine provocation in asthma/allergic airway disease mouse models are the Scireq flexiVent, eSpira EMMS Spirometry, and Buxco FinePointe Resistance and Compliance systems. Asthma/allergic airway disease models routinely use the Scireq flexiVent system to perform perturbations that assess Newtonian Resistance/central airways resistance (Rn), tissue elastance (H; measure of alveolar tissue stiffness) and damping (G; index of alveolar tissue restriction), and/or transpulmonary resistance (Rrs), elastance (Ers), and compliance (Crs), following provocation with nebulized methacholine (up to 100 mg/kg) (Kim et al., 2017a; Kim et al., 2017b; Piyadasa et al., 2018; Ambhore et al., 2019; Kalidhindi et al., 2019; Ali et al., 2020a; Pinkerton et al., 2022; Tu et al., 2022; Xiao et al., 2022). Increases in Rn, H, G, Rrs, and Ers, and decreased Crs, when compared to control (non-allergic) mice is commonly reported. Whilst the order and timing of perturbations is usually dependent on the stimulus used to induce asthma/allergic airways disease, the tidal volume is commonly set at between 8 and 10 mL/kg and the respiratory rate at 150 or 450 breaths/minute. The Buxco invasive plethysmograph also measures airway resistance following provocation with nebulized methacholine (up to 8 mg/kg) (Royce et al., 2011; Donovan et al., 2013; Royce et al., 2022), however, this system does not differentiate between central airways and transpulmonary measurements. Nevertheless, invasive plethysmography measures key features of AHR in response to provocation in experimental asthma models and provides sensitive detection of changes in lung function parameters that are highly representative of those that occur in patients with asthma and severe asthma.

### 1.5 *Ex vivo* techniques to assess AHR

AHR and its driving mechanisms can also be assessed using specialized *ex vivo* techniques. Airway tissues, such as bronchi and trachea, can be isolated from human lung resections or dissected from mice, and airway reactivity assessed to contractile stimuli in organ bath or myograph systems. Trachea isolated from mouse models of experimental asthma exhibit AHR to methacholine, which is a similar assessment to invasive plethysmography techniques (Donovan et al., 2013) and is a platform to assess airway reactivity *ex vivo*. Recent advances in human and mouse PCLS preparations have also allowed for assessment of AHR *ex vivo*, with PCLSs being able preserve the natural tissue architecture and interdependency of the ASM and the surrounding parenchyma. PCLS have also been used to assess changes in small airway reactivity and calcium signaling within ASM cells in response to contractile and bronchodilator stimuli (Donovan et al., 2013; Bourke et al., 2014; Donovan et al., 2015; Liu et al., 2017). Application of this technique in mouse models of experimental asthma has provided significant insights that complement and advance studies on isolated larger airways, where airway inflammation has been shown to alter airway reactivity (Sukkar et al., 2001; Tliba et al., 2003; Zhang et al., 2007). PCLSs from house dust mite antigen (HDM)-treated mice have increased contraction to methacholine (measured as a reduction in airway lumen area) compared to PBS control-treated mice (Liu et al., 2017). In contrast, PCLSs from ovalbumin (ova)-treated mice have reduced methacholine-induced contraction despite having increased AHR when measured *in vivo* and in isolated trachea (Donovan et al., 2013). Whilst this technique provides a unique platform to assess AHR and calcium signaling in ASM, it is important to consider that airway stiffness may impair airway contraction and confound assessment of AHR in models with robust airway remodeling.

### 1.6 *In vitro* techniques to assess AHR

It is possible to examine and measure some biologically relevant properties of ASM in isolated cell culture systems, such as changes in calcium signaling using calcium dyes (Bourke et al., 2011) and its hypercontractile properties using collagen gel preparations (as a surrogate for contraction) (Matsumoto et al., 2007; Bourke et al., 2011). The properties of ASM *in vitro* can be affected by different culture conditions, however it is now well accepted that isolated ASM cells from patients with asthma have altered contractile, proliferative and secretory function compared to those from subjects without asthma [reviewed in (Zuyderduyn et al., 2008)]. ASM from patients with asthma have increased contraction to histamine compared to ASM from subjects without asthma as measured by a decrease in collagen gel area (Matsumoto et al., 2007). However, whether these properties of ASM are also observed in cells isolated from mouse models of asthma remains to be determined and may provide a new platform to accelerate drug discovery. Lung-on-a-chip technology allows *in vitro* modeling and study of human musculature and may be a feasible approach for such studies. Engineered human airway musculature can recapitulate healthy and asthmatic bronchoconstriction and bronchodilation. Inhibition of Rho-mediated calcium sensitization and contraction in this model has been shown to decrease basal tone of bronchial smooth muscle and prevent hypercontraction (Nesmith et al., 2014).

## 2 Mouse models of experimental asthma

Murine models of experimental asthma facilitate the investigation of mechanisms underpinning disease pathogenesis, the identification of novel therapeutic targets, and are platforms for evaluating the efficacy and safety of novel therapeutics before they enter clinical trials. However, mice do not develop asthma spontaneously and this necessitates the experimental induction of allergic responses in the airways to model this disease. Numerous models have been developed and utilize different allergens (ova, HDM, cockroach, pollen), routes of allergen administration (intraperitoneal, subcutaneous, intranasal, aerosol inhalation), and exposure protocols (single *versus* multiple consecutive exposures to allergen). Despite these differences, the principles of inducing experimental asthma are common and typically involve an initial allergen sensitization phase followed by a challenge phase where mice are re-exposed to the sensitizing agent to initiate allergic recall. These models have been successfully developed in multiple strains of mice, however, BALB/c strain mice are used most often owing to their T_H_2-prone immunological response, which allows these models to more accurately recapitulate hallmark features of allergic asthma including airway inflammation, airway remodeling, and AHR. Importantly, the presence and severity of these features are dependent on the type of allergen used to induce disease, the exposure protocol, and the measurement of AHR recorded (Table 1). In addition, the choice of equipment used to measure AHR can result in different outcomes, mainly in relation to airway resistance, in different regions of the lung (Rn, Rrs, airway resistance). Nevertheless, invasive techniques (e.g., Scireq flexiVent, Buxco systems and eSpira EMMS Spirometry) do provide more accurate recordings of resistance than non-invasive techniques (*e.g.*, enhanced pause [Penh]) [comprehensively reviewed in (Bates and Irvin, 2003)]. Comparing AHR across experimental groups in different studies is challenging as the measurements of AHR are not directly interchangeable due to the equipment used and often mice have different baseline levels of resistance depending on the calibration of the machines. In some models presented in Table 1, the data are expressed as % change from baseline/saline which does allow for direct comparisons between experimental groups and different experiments in other publications. For transparency in future studies, presenting % change and raw data would allow for readers to directly compare the degree of AHR induced by different exposure protocols to determine the most appropriate model of experimental asthma to employ. This, along with strain, allergen and exposure protocol, is imperative to carefully consider the outcomes of interest against the endo/phenotype that each model can offer.

**TABLE 1:** Experimental asthma models.

Allergen/strain/sex	Method of administration	Model protocol	Disease features	Ref
Ova BALB/c female	Sensitization: IP challenge: IN	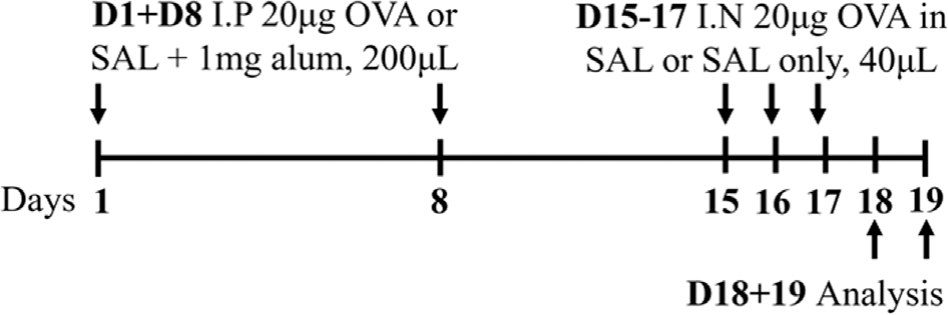	*C.f.* Saline IN	Kim et al. (2019)
			↑ AHR (MCh; Rrs and elastance; eSpira, EMMS)	
			↑ Eosinophils	
			↔Neutrophils	
Ova BALB/c female	Sensitization: IP challenge: NEB 5% (v/v) 30 min	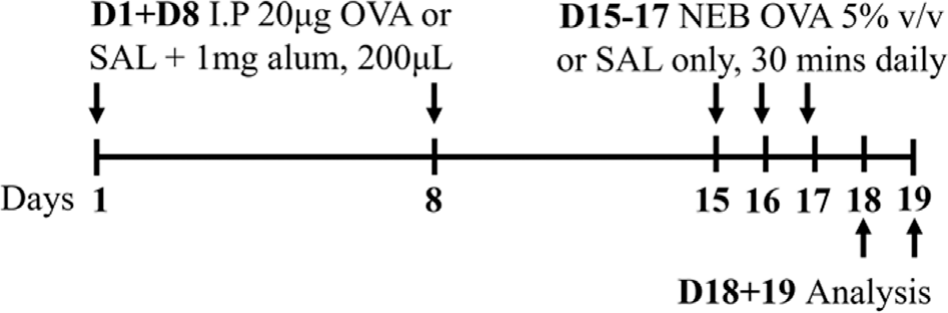	*C.f.* Saline aerosolized	Kim et al. (2019)
			↑ AHR (MCh; Rrs and elastance; eSpira, EMMS)*	
			↑ Eosinophils	
			↑ Neutrophils	
			* = *C.f* Ova IN	
Ova BALB/c female	Sensitization: IP challenge: IN	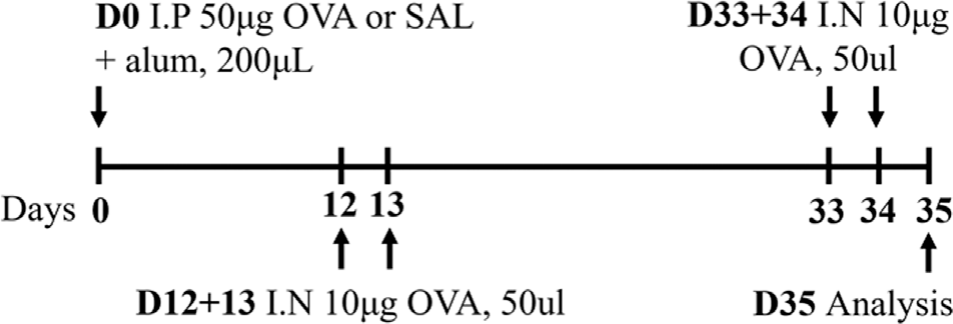	*C.f.* Saline	Essilfie et al. (2015), Kim et al. (2017a), Kim et al. (2017b)
			↑ AHR (MCh; Rn; Scireq flexiVent)*	
			↑ Eosinophils	
			↑ Neutrophils	
Ova BALB/c or C57BL6J/129SV female	Sensitization: IP challenge: NEB 2.5% (w/v)	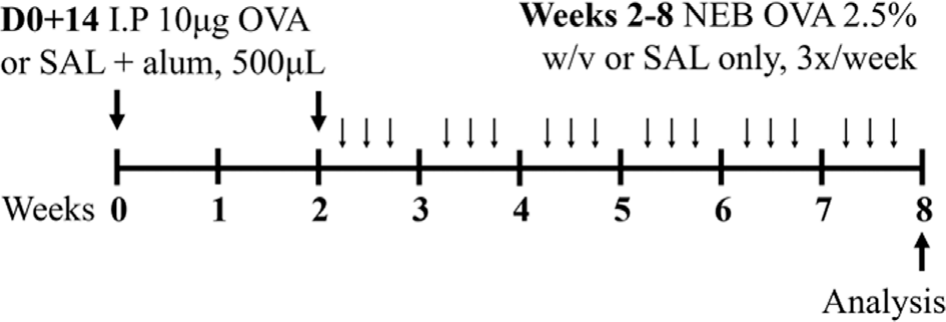	*C.f.* Saline	Mookerjee et al. (2006), Royce et al. (2011)
			↑ AHR (MCh; resistance; Buxco system)	
			↑ Leukocytes	
			↑ Eosinophils	
			↑ Neutrophils	
			↑ Collagen	
			↑ Goblet cell hyperplasia	
Ova BALB/c female	Sensitization: IP challenge: NEB 2.5% (w/v)	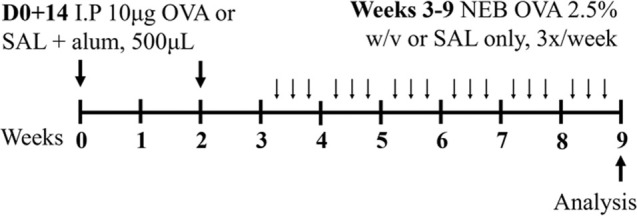	*C.f.* Saline	Donovan et al. (2013), Royce et al. (2022)
			↑ AHR (MCh; resistance; Buxco system)	
			↑ Leukocytes	
			↑ Eosinophils	
			↑ Neutrophils	
			↑ Collagen	
			↑ Goblet cell hyperplasia	
HDM BALB/c female	Sensitization: IN challenge: IN	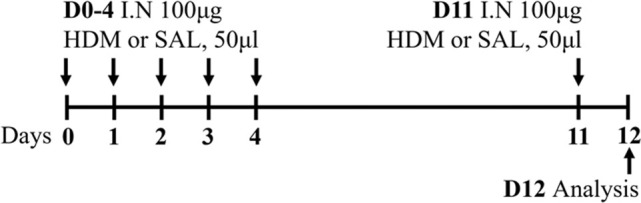	*C.f.* Saline	Woo et al. (2018)
			↑ AHR (MCh; Rn and Rrs; Scireq flexiVent)	
			↑ Neutrophils	
			↑ Goblet cell hyperplasia	
HDM BALB/c female	Sensitization: IN challenge: IN	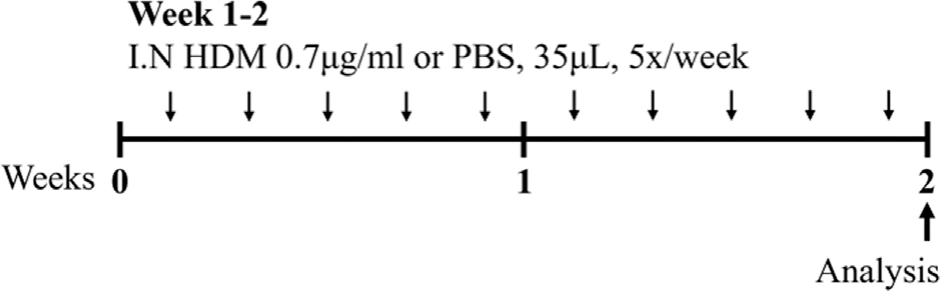	*C.f.* Saline	Piyadasa et al. (2018)
			↑ AHR (MCh; Rn, damping and elastance; Scireq flexiVent)	
			↑ Leukocytes	
			↑ Eosinophils	
			↑ Neutrophils	
HDM BALB/c male	Sensitization: IN challenge: IN	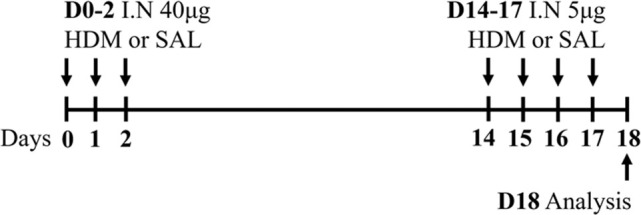	*C.f.* Saline	Barry et al. (2013)
			↑ AHR (MCh; resistance; Buxco system)	
			↑ Eosinophils	
			↑ Neutrophils	
			↑ Goblet cell hyperplasia	
HDM BALB/c female	Sensitization: IN challenge: IN	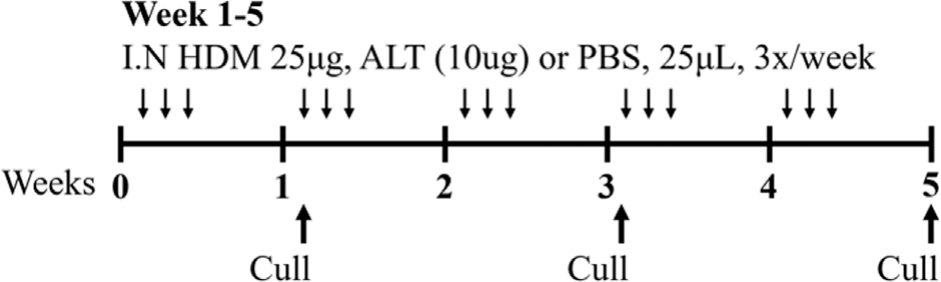	*C.f.* PBS	Uwadiae et al. (2019)
			↑ AHR (MCh; airway resistance and elastance; Scireq flexiVent)	
			↑ Lung cells (+ eosinophils) by flow cytometry	
HDM BALB/c female	Sensitization: IN challenge: IN	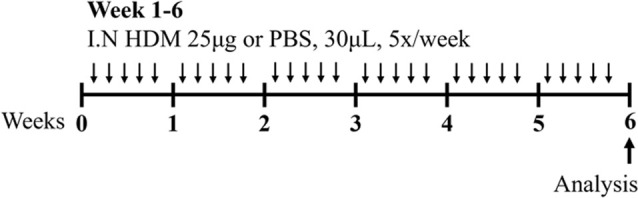	*C.f.* PBS	Liu et al. (2017), Ali et al. (2020a)
			↑ AHR (MCh; Rn; Scireq flexiVent or resistance; Buxco system)	
			↑ Leukocytes	
			↑ Collagen	
			↑ Goblet cell hyperplasia	
HDM BALB/c female	Sensitization: IN challenge: IN	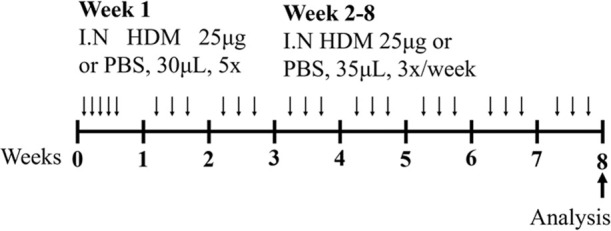	*C.f.* Saline	Woo et al. (2018)
			↑ AHR (MCh; Rn and Rrs; Scireq flexiVent)	
			↑ Leukocytes*	
			↑ Eosinophils	
			↑ Neuts**	
			*C.f. 4-week chronic model	
			**C.f. 4-week chronic model and 2-week acute model	
HDM BALB/c female	Sensitization: IN challenge: IN	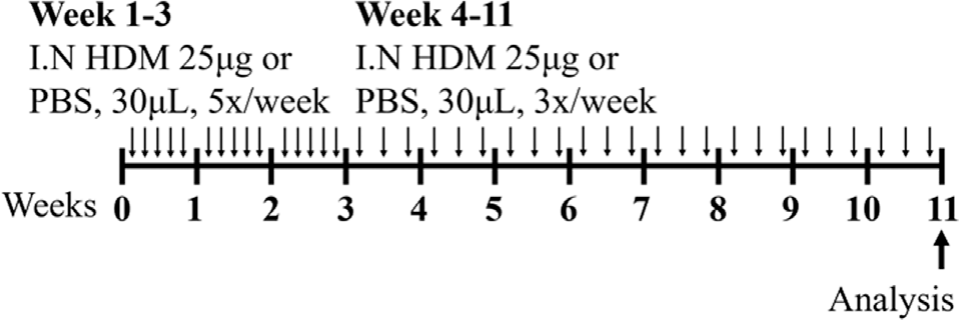	*C.f.* PBS	Tu et al. (2022)
			↑ Leukocytes	
			↑ Eosinophils	
			↑ Neutrophils	
			↑ Goblet cell hyperplasia	
			↑ AHR (MCh; Rn; Scireq flexiVent)	
Cockroach C57Bl/6 sex not specified	Sensitization: IN challenge: IN	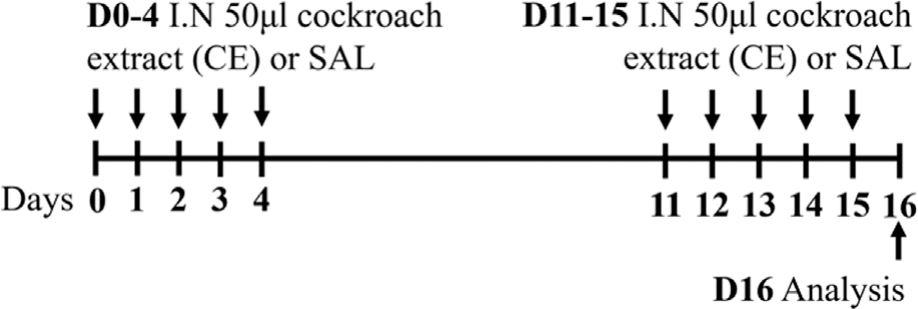	*C.f.* Saline	Alqarni et al. (2022)
			↑ Leukocytes	
			↑ Eosinophils	
			↑ Leukocytes	
			↑ Mucus secretion (Muc5ac increased)	
Cockroach BALB/c male	Sensitization: IN challenge: IN	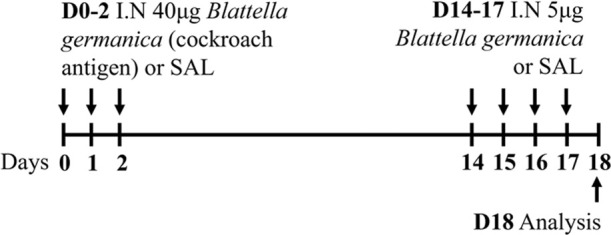	*C.f.* Saline	Barry et al. (2013)
			↑ Eosinophils	
			↑ Goblet cell hyperplasia	
			↑ AHR (MCh; resistance; Buxco system)	
Pollen BALB/c female	Sensitization:SC challenge: NEB	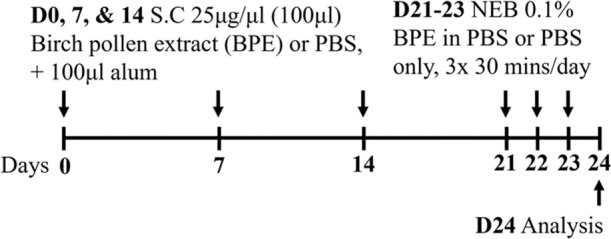	*C.f.* Saline	Xie et al. (2021)
			↑ Eosinophils	
			↑ Neutrophils	
			↑ Inflammatory score	
Pollen BALB/c female	Sensitization: IP challenge: NEB	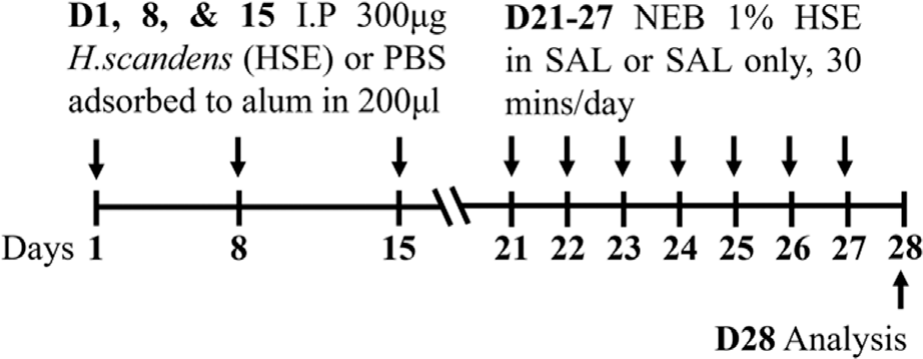	*C.f.* PBS IP	Xi et al. (2021)
			↑ AHR (MCh; enhanced pause [Penh])	
			↑ Leukocytes	
			↑ Eosinophils	
			↑ Neutrophils	
			↑ Inflammatory scores	
			↑ Goblet cell hyperplasia	
Pollen BALB/c female	Sensitization:SC challenge: NEB	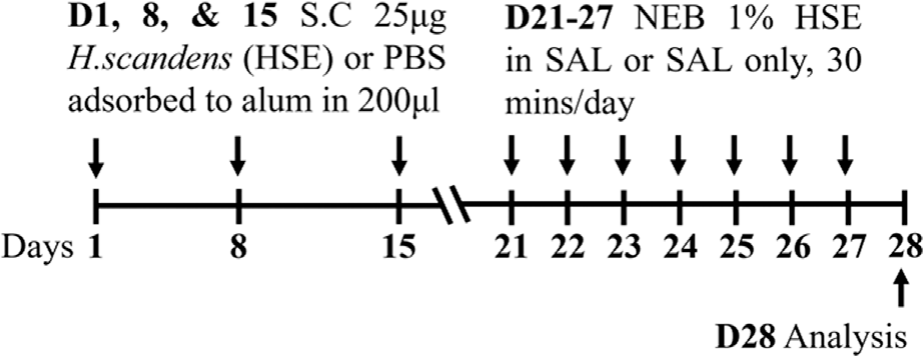	*C.f.* PBS SC	Xi et al. (2021)
			↑ AHR (MCh; Penh)	
			↑ Leukocytes	
			↑ Eos*	
			↑ Neutrophils	
			↑ Inflammatory scores	
			↑ Goblet cell hyperplasia	
			*C.f. HSE IP	
Fungal spores BALB/c sex not specified	Sensitization: IN (spores) challenge: IN (spores)	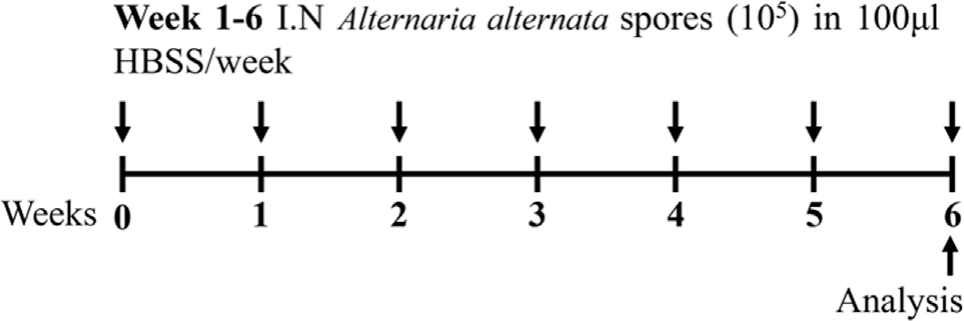	*C.f.* HBSS control	Daines et al. (2021)
			↑ AHR (MCh; resistance; Scireq flexiVent)	
			↑ Leukocytes	
			↑ Eosinophils	
			↑ Neutrophils*	
			↑ Inflammatory scores	
			↑ Goblet cell hyperplasia	
			*C.f. Filtrate model	
Fungal spores BALB/c sex not specified	Sensitization: IN (filtrate) challenge: IN (filtrate)	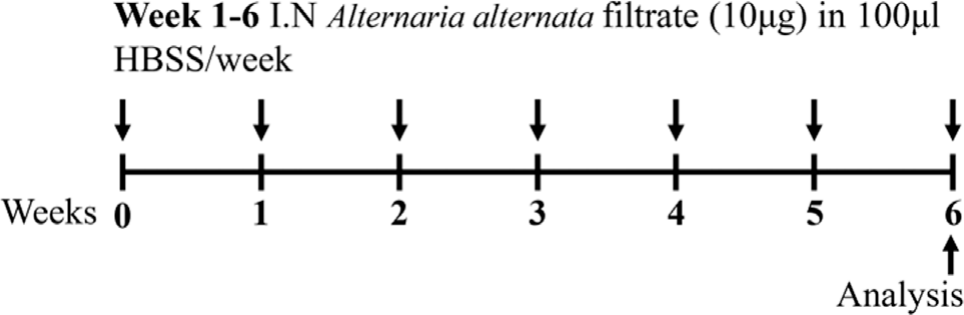	*C.f* filtrate control	Daines et al. (2021)
			↑ AHR (MCh; resistance; Scireq flexiVent)	
			↑ Leukocytes	
			↑ Eosinophils*	
			↑ Neutrophils	
			↑ Inflammatory scores	
			↑ Goblet cell hyperplasia	
			*C.f. Spores model	

### 2.1 Ovalbumin-induced mouse models of experimental asthma

The mostly widely used allergen that is used to induce experimental asthma is ova, derived from chicken egg protein. Ova is commonly administered systemically, via intraperitoneal or subcutaneous injection, in conjunction with the potent T_H_2-inducing adjuvant, aluminium hydroxide (Royce et al., 2011; Donovan et al., 2013; Essilfie et al., 2015; Kim et al., 2017b; Kim et al., 2019; Pinkerton et al., 2022) to drive allergic sensitization. This is followed at a later time by respiratory challenge with ova to initiate allergic recall responses and model allergen-induced exacerbations of established allergic disease. These challenges are often administered intranasally; however, a recent study highlighted that aerosol challenge, compared to intranasal challenge with ova, induces greater AHR in response to 50 mg/mL dose of methacholine suggesting that ova-induced experimental asthma responses are more potently induced by inhaled, rather than intranasal, ova challenge (Kim et al., 2019). Ova-induced models are renowned for their reproducibility and robust allergic inflammatory responses that recapitulate key disease features including airway inflammation, epithelial hypertrophy, goblet cell hyperplasia, and AHR (Essilfie et al., 2015; Kim et al., 2017b; Kim et al., 2019; Pinkerton et al., 2022). However, and despite this favorable set of outcomes for modeling asthma experimentally, there is contention about the clinical relevance of ova as an allergen, which is an important limitation of this model as it highlights a potential barrier for translation of findings into human disease. In addition, allergen sensitization via intraperitoneal injection in conjunction with an adjuvant does not reflect the natural course of allergen sensitization in asthma in humans. Ova models are also not associated with persistent airway remodeling, which is a key feature of human asthma, and the chosen allergen exposure regimen may induce disease features that are, or are not, sensitive to corticosteroid treatment, which further highlights potential limitations of ova-based asthma models. More recently, experimental asthma models have moved towards utilizing more clinically and physiologically relevant allergens and model protocols that are strongly linked with the human disease.

### 2.2 HDM-induced mouse models of experimental asthma

Sharing many similarities to human disease, mouse models of HDM-induced experimental asthma are characterized by eosinophil-dominant airway inflammation, airway remodeling, and AHR (Barry et al., 2013; Woo et al., 2018; Ali et al., 2020a; Tu et al., 2022). The immune response to HDM is also T2-driven, with increased IL-5, IL-13, IL-33, IL-25, and thymic stromal lymphopoietin contributing to eosinophils, type 2 innate lymphoid cells (ILC2s) and dendritic cell recruitment/activation in the lung [reviewed in (Jacquet, 2021)]. The hallmark features of disease are similar to ova-induced experimental asthma, however HDM-induced experimental asthma can be considered more clinically relevant as these models have allergic sensitization through the intranasal route, which can be achieved in the absence of an adjuvant and is a more realistic representation of what occurs in humans. Furthermore, HDM induces allergic airways disease through a combination of toll-like responses and enzymatic reactions [reviewed in (Abu Khweek et al., 2020)], which may be absent in ova models. The specific protocol of HDM antigen exposure is also an important consideration; with acute vs*.* chronic HDM exposure models leading to different disease outcomes (Woo et al., 2018) (Table 1). Furthermore, multiple preparations of HDM antigen can be used as an allergen (whole extract vs*.* components) and this represents an important limitation in published studies to date, where the type of HDM preparation that is used for experimentation is often not fully specified. In particular, the batch numbers of HDM allergens used, and whether the HDM protein concentration is determined by bicinchoninic acid assay (BCA) or Bradford assessment, is often omitted from manuscripts.

### 2.3 Fungal-induced mouse models of experimental asthma

Another common environmental aeroallergen is fungus. Fungal asthma is poorly managed and causes frequent exacerbations and hospitalizations (Lehmann et al., 2017). Fungal asthma is considered predominately a T2 driven disease, however, it can be associated with T2 low endotypes of asthma (T_H_1 and T_H_17 high) (reviewed in [van Tilburg Bernardes et al., 2020)]. *Alternaria alternata* is an allergenic mold that is recognized as a potent aeroallergen and a common inducer of asthma exacerbations (Daines et al., 2021). When employed in murine models of fungal asthma, filtrates of *A. alternata* are used to sensitize and challenge mice. Recent studies have also shown that direct spore exposure, as opposed to traditional filtrate exposure, is more physiologically relevant and has the capacity to induce similar (and more potent) disease outcomes (Daines et al., 2021) (Table 1). *Aspergillus fumigatus* has also been used in combination with allergen/s to mimic disease outcomes. The double allergen model involves sensitization and challenge with dust mite (*Dermatophagoides farinae*) followed by intranasal exacerbation with *A.* fumigatus (Matsuse et al., 2013). This model has demonstrated key features of experimental asthma, including increased inflammation (macrophages, neutrophils, eosinophils and lymphocytes, IL-5, IL-13), and mucus secreting cells, and partial steroid-insensitivity to dexamethasone (decreased macrophages, eosinophils, and IL-5, but no effect on neutrophils, lymphocytes, IL-13, mucus-secreting cells) (Matsuse et al., 2013). Furthermore, triple allergen models of experimental asthma have been established to more accurately recapitulate the human scenario. These models involve sensitization (on day 0 and 5) with a triple allergen cocktail (dust mite [*D. farinae*], ragweed [*Ambrosia artemisiifolia*], and *A. fumigatus*) in the presence of an adjuvant, followed by intranasal administration of the triple allergen mixture on days 12, 13, and 14 (Chung et al., 2020). This model induces hallmark features of asthma including inflammation, remodeling (increased mucus secreting cells) and AHR (increased resistance to MCh; Scireq flexiVent).

The continual optimization of murine models of experimental asthma is an important and indispensable paradigm in the journey towards increasing our understanding of the underlying physiological mechanisms that drive asthma pathogenesis. Crucially, to address the heterogeneous nature of asthma, researchers have developed many different allergen-induced models that have allowed for recapitulation of different endotypes and phenotypes of asthma. Consequently, this has led to the development of experimental protocols that model severe, steroid insensitive or resistant phenotypes of asthma; allowing for the identification of mechanisms that may drive the onset of more severe disease.

### 2.4 Infection-induced mouse models of experimental severe asthma

Mouse models of experimental severe asthma that recapitulate features of human disease have provided invaluable insight into the possible mechanisms that are responsible for driving the increased disease severity and steroid insensitivity that are characteristic of severe asthma in humans (Table 2). Severe asthma is often characterized by an insensitivity to currently available therapeutics and the capacity to model this association experimentally is crucial. Improving our understanding of mechanisms underpinning steroid insensitivity (sometimes referred to as steroid resistance in publications) will identify novel targets for therapeutic development. Importantly, there is strong evidence that supports a link between specific respiratory infections and severe asthma exacerbations, whereby different infections can induce different phenotypes of severe disease (e.g., neutrophilic-dominant vs*.* eosinophil-dominant). Recent advances in murine models have incorporated these elements into the protocol design and have been optimized to model and investigate the effect of respiratory infections on disease outcomes (Essilfie et al., 2012).

**TABLE 2:** Experimental mixed-exposure asthma models.

Stimulus/Allergen/Strain/Sex	Method of administration	Model protocol		Disease features	Ref
LPS/HDM BALB/c sex not specified	Sensitization: IP challenge: IN	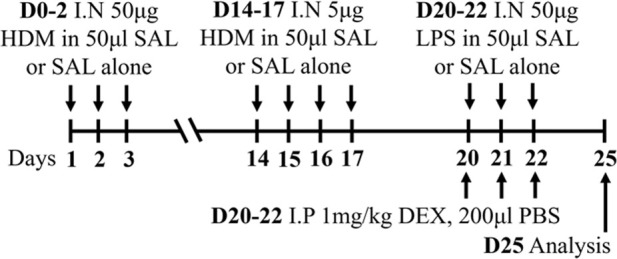		*C.f.* HDM or LPS alone	Wang et al. (2021a)
				↑ AHR (MCh; Rn; Scireq flexiVent)*	
				↑ Airway inflammation*	
				↑ Goblet cell hyperplasia	
				↑ Inflammatory scores	
				* Steroid insensitive	
NTHi/Ova BALB/c female	Sensitization: IP challenge: IN	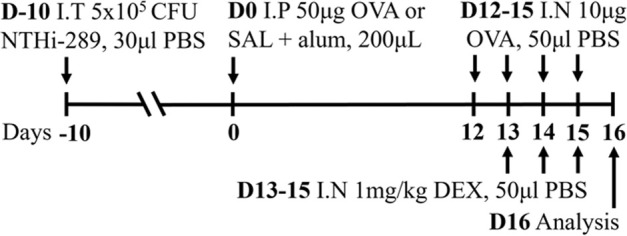		*C.f.* Ova alone	Essilfie et al. (2012), Essilfie et al. (2015)
				↔AHR (resistance; Buxco system)*	
				↔ Total airway inflammation	
				↓ Eosinophils*	
				↑ Neutrophils*	
				* Steroid insensitive	
Cmu/Ova BALB/c female	Sensitization: IP challenge: IN	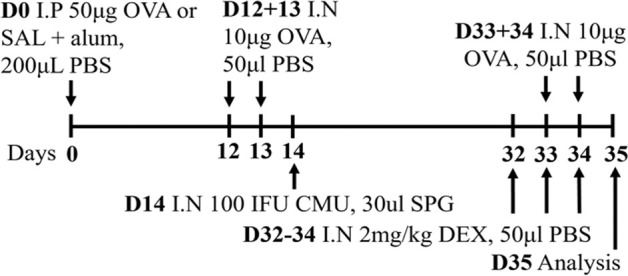		*C.f.* Ova alone	Essilfie et al. (2015), Kim et al. (2017a), Kim et al. (2017b)
				↑ Total airway inflammation*	
				↓ Eosinophils	
				↑ Neutrophils*	
				↑ Macrophages*	
				↔ AHR (Rn; Scireq flexiVent)*	
				* Steroid insensitive	
RSV/Ova BALB/c male	Sensitization: IP challenge: IN	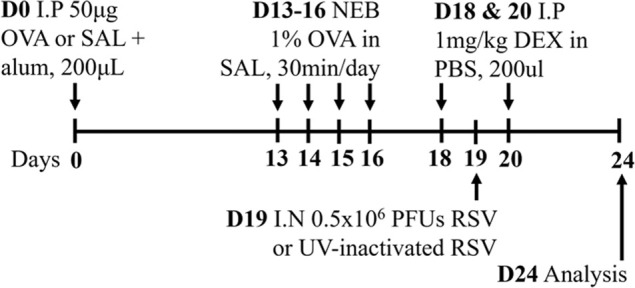		*C.f.* Ova alone	Nguyen et al. (2016a), Nguyen et al. (2016b), Nguyen et al. (2018)
				↔AHR (MCh; (Rn; Scireq flexiVent)* (*partially steroid resistant)*	
				↑ Neutrophils*	
				↔ Eosinophils*	
				* Steroid insensitive	
RSV/Ova BALB/c female	Sensitization: IP challenge: IN	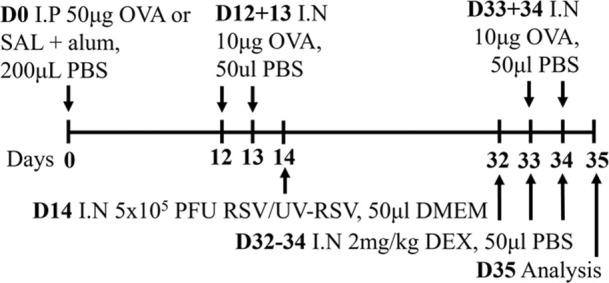		*C.f* Ova alone	Kim et al. (2017a)
				↔AHR (Rn; Scireq flexiVent) (methacholine)*	
				↔ Neutrophils*	
				↔ Eosinophils*	
				* Steroid insensitive	
RSV/HDM C57Bl/6 male	Sensitization: IN challenge: IN	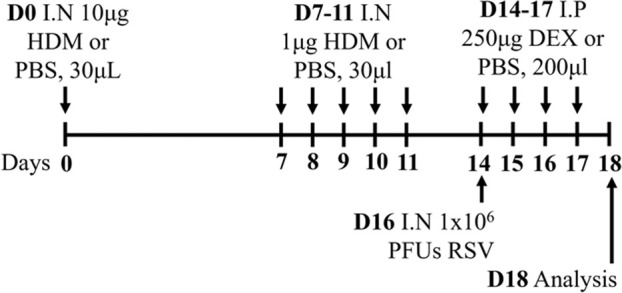		*C.f* HDM alone	Makino et al. (2021)
				↑ AHR (MCh; Rrs; Scireq flexiVent)*	
				↑ total airway inflammation	
				↔ Eosinophils*	
				↑ Neutrophils*	
				* Steroid insensitive	
HDM/cyclic-di-GMP BALBc/ByJ male and female	Sensitization: IN challenge: IN	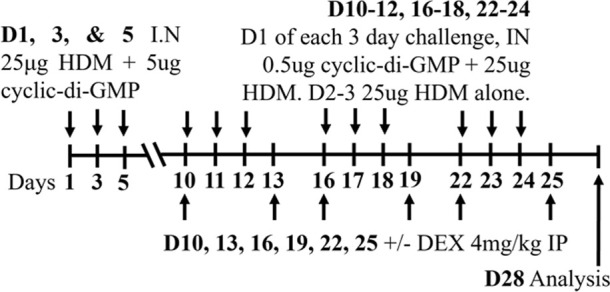		*C.f.* HDM alone	Raundhal et al. (2015), Oriss et al. (2017), Gauthier et al. (2022)
				↑ AHR (MCh; Rn or Rrs; Scireq flexiVent)*	
				↑ Neutrophils*	
				↔ total airway inflammation*	
				* Steroid insensitive	

Infectious pathogens such as *C. pneumoniae* (Essilfie et al., 2015; Kim et al., 2017a; Kim et al., 2017b), *H. influenzae* (Essilfie et al., 2012; Essilfie et al., 2015; Kim et al., 2017a), RSV (Nguyen et al., 2016a; Nguyen et al., 2016b; Kim et al., 2017a; Nguyen et al., 2018), influenza A virus (Kim et al., 2017a), and rhinovirus (Bartlett et al., 2008) have been used to induce severe disease in mouse models of experimental asthma. These infections are often induced through natural respiratory routes after experimental asthma is established, which reflects the typical scenario of an infection-induced exacerbation of asthma. An important element of these infection-induced models is their capacity to model different phenotypes of severe disease, which provide a platform for the elucidation of broadly applicable and/or phenotype-specific mechanisms of pathogenesis. When infections with the natural mouse respiratory pathogens *Chlamydia muridarum* or non-typeable *H. influenzae* are induced in female BALB/c mice with ova-induced experimental asthma, this results in steroid-insensitive disease (AHR and total airway inflammation that are insensitive to dexamethasone treatment) with a switch from eosinophilic to neutrophil-dominant airway inflammation (Kim et al., 2017a). Importantly, these preclinical observations are validated by clinical observations that show an association between bacterial infection and neutrophil-dominant inflammation in the sputum of patients with asthma (Wood et al., 2010). Contrastingly, whilst viral infection (mouse-adapted influenza A virus or RSV), like with bacterial infection-induced models of severe asthma, also induce steroid-insensitive AHR, there is no switch towards neutrophil-dominant inflammation and total airway inflammation becomes partially steroid-insensitive (Kim et al., 2017a). Rhinoviruses are also important viral infections involved the increased risk of asthma and asthma exacerbations; however, mouse models of rhinovirus infections are challenging as the major group of rhinoviruses (that comprises 90% of all rhinovirus serotypes) requires human intercellular adhesion molecule-1 (ICAM-1) for replication, which is not present in mice. Nevertheless, minor group rhinoviruses, such as rhinovirus-1B, can replicate in wild-type BALB/c mice, and this has been shown to increase AHR in ova-induced experimental asthma associated with increased neutrophil responses (Bartlett et al., 2008). Whether this model of rhinovirus and ova-induced experimental asthma is steroid-insensitive is yet to be elucidated.

Viral infection models of severe asthma are characterized by steroid-insensitive eosinophilic airway inflammation and AHR (Kim et al., 2017a), and this agrees with previous studies showing that eosinophilic inflammation can be present despite moderate-to high-dose steroid treatment (Jayaram et al., 2005; Trevor and Deshane, 2014). Interestingly, in infection-induced models (bacterial and viral), aberrant microRNA-21 (miR-21) responses have been shown to drive steroid insensitivity in experimental severe asthma and therapeutically targeting miR-21 may offer a broadly effective treatment for infection-induced severe, steroid-insensitive asthma.

In addition, components of bacteria, such as lipopolysaccharide (LPS) or cyclic-di-GMP, have been utilized in HDM-induced models of experimental asthma to induce features of severe asthma (Raundhal et al., 2015; Gauthier et al., 2017; Oriss et al., 2017; Wang et al., 2021a; Gauthier et al., 2022). These models have provided significant mechanistic insight into the clinical observation that patients with severe asthma, particularly T2 low patients, are steroid-insensitive (Woodruff et al., 2009). Importantly, these studies together have identified key disease-causing and interconnected roles of dysregulated CXCR3 ligand (CXCL10)/T_H_1 cell recruitment/IFNγ/secretory leukocyte protease inhibitor (SLPI) responses in steroid-insensitive severe asthma (Raundhal et al., 2015; Gauthier et al., 2017; Oriss et al., 2017; Gauthier et al., 2022).

Collectively, infection- or bacterial component-induced models of severe asthma continue to provide important mechanistic insight into disease pathogenesis whilst also highlighting the complexity of severe disease. Considering the growing appreciation for the differences in immune responses between male and females, and the differences between sex hormones, genetics, and epigenetics in asthma [reviewed in (Chowdhury et al., 2021)], an important future direction will be to understand how sex hormones influence disease outcomes in these models. The role/s of sex hormones influencing asthma and AHR is yet to be fully elucidated however, there is observational data demonstrating that estrogen receptor *β* knockout male and female mice have increased AHR in a mixed allergen (ovalbumin, *A. alternata*, *A. fumigatus*, and *D. farinae*) model compared to wild type mixed allergen alone (Kalidhindi et al., 2019), however the specific roles of hormone receptors in asthma and the magnitude of AHR remains to be fully explored. It will also be important to determine how obesity influences immune responses to infection, both alone and in the context of asthma and AHR, and to understand whether the pathogenic mechanisms that have been identified in models of infection-induced disease (e.g., miR-21) are also relevant in obesity-associated disease.

### 2.5 Asthma-chronic obstructive pulmonary disease (COPD) overlap

Asthma can also overlap with COPD in patients, and these patients are currently referred to as having asthma-COPD overlap (ACO). Similar to severe asthma, patients with ACO have chronic airway inflammation that is linked with airway remodeling, decreased lung function, wheezing, and shortness of breath (Leung and Sin, 2017). The cellular composition of the airway inflammation in these patients is also heterogeneous, however, ACO has similar endotypes to severe asthma (T2 high, T2 low, or non-T2). Recent advances in mouse models of experimental ACO [reviewed in (Tu et al., 2021)] have provided important mechanistic insights into novel, disease-causing mechanisms in ACO and identified potential therapeutic targets that may also be applicable to other forms of asthma. Experimental ACO can be induced by chronic administration of HDM antigen and concurrent exposure to cigarette smoke after HDM-induced experimental asthma is established (Tu et al., 2022). Importantly, this model results in steroid insensitivity of AHR and other key disease features such as airway remodeling, which is reminiscent of ACO in humans (Dey et al., 2022). Comprehensive transcriptomic analysis of airway and parenchyma tissues in this model revealed robust increases in innate immune responses (increased SPI1, TFEC), complement responses, and disturbances in the expression of metabolism-associated factors (increased GLMP, PLSCR1, MTF1) (Tu et al., 2022). Significantly, therapeutic targeting of SPI1 suppressed some of the key features of experimental ACO, including steroid insensitive AHR (Tu et al., 2022). These data show that the combination of HDM antigen and cigarette smoke exposures drive changes in lung function that are not responsive to mainstay therapies and highlight the potential utility of this model for deciphering the specific mechanisms that cause this outcome.

### 2.6 High fat diet-induced experimental obesity asthma models

Obesity has been linked with the development of asthma, however, the mechanisms underpinning this relationship remain largely unknown. The obese asthma phenotype represents a significant cohort of asthmatics who have more severe disease that is commonly refractory to mainstay therapies (Dixon et al., 2011). To improve our understanding of obese asthma and the mechanisms underpinning the severe, steroid insensitivity of this disease phenotype, multiple mouse models of obesity-associated asthma have been developed and optimized.

Obesity can be modeled in mice through genetic modifications (mono, polygenic mutations, transgenic mice), surgical or chemical induction, or, more commonly, by the administration of hypercaloric, specialized diets (Pinkerton et al., 2022). Diet-induced models of obesity lead to dietary imbalance, which is the most common cause for obesity in humans. Compared with genetically induced/modified models of obesity, the timeframe of diet-induced obesity is often protracted to model the natural and gradual accumulation of adiposity that is observed in obesity in humans. A range of different diets have been recognized to induce obesity in mice, including high-fat diets (HFD), high-sugar diets, and a combination of the two that represents a Western diet (high fat and high sugar). Whilst this array of diets allows for different facets of obesity and metabolic disorders to be modeled, it is important to consider the variations in the macro- and micronutrient profiles of these different diets. This variability highlights the lack of a standardized, well-defined diet for modeling obesity and may underpin the discrepancies in reported outcomes between studies. Therefore, to ensure reproducibility and consistency across investigations, it is important that the formulation of diets that are used to induce obesity are considered and specified.

These different models of obesity have facilitated investigations into the complex relationship between obesity and asthma (Kim et al., 2014; Pinkerton et al., 2022). HFD-induced obesity in mice (HFD; 60% energy derived from fat; Research Diets, D12492) is characterized by increased total body mass, inflammation (measured in terms of increased macrophages and ILC3s in the lung and increased macrophages in adipose tissues), and AHR in response to methacholine provocation compared to control chow (CC)-fed mice (Kim et al., 2014). Importantly, this obesity-induced AHR has been associated with NLRP3 and IL-17 pathway-dependent IL-17^+^ILC3 responses. In an independent and more recent study, HFD-induced obesity in mice (HFD; 60% energy derived from lipids, 15% energy from protein; Specialty Feeds, SF14-154), was also shown to be characterized by increased total body mass as well as adiposity where robust increases in the mass of major white adipose tissue pads were observed, including the parametrial, inguinal, and retroperitoneal fat pads compared to CC-fed mice (CC; 16% energy derived from lipids, 21% energy from protein; Specialty Feeds SF09-091; Table 3) (Pinkerton et al., 2022). Furthermore, when superimposed with ova-induced experimental asthma, HFD-induced obesity was shown to promote steroid-insensitive AHR but with no significant effect on allergic airway inflammation when compared to CC-fed, allergic mice. Significantly, this study also highlighted a novel association between increased T2 immune (concomitant IL-5 and IL-13 responses) and NLRP3 inflammasome responses in the airway in obesity-associated severe asthma, providing a potential mechanism and explanation as to why obese individuals experience more severe asthma that is refractory to current therapies (Pinkerton et al., 2022). Importantly, the pre-clinical findings from this study have been validated in clinical samples, demonstrating this model’s utility for increasing the understanding of the mechanistic interplay underpinning obesity-associated severe asthma in humans. However, whether therapeutic targeting of T2-induced, NLRP3-mediated responses in obesity-associated severe asthma is effective in humans, remains to be determined.

**TABLE 3:** Experimental asthma models (diet).

Allergen	Method of administration	Model protocol	Disease features	Ref
HFD/OVA BALB/c female	Sensitization: IP challenge: IN	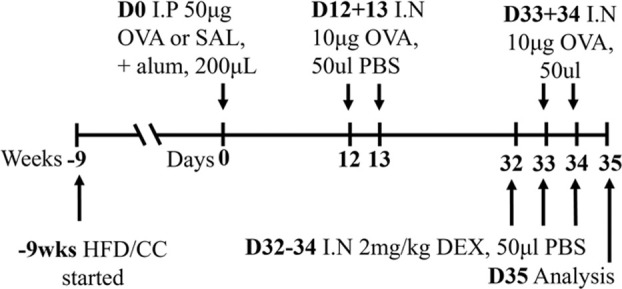	C.f. CC/OVA (lean disease)	Pinkerton et al. (2022)
			↔AHR (MCh)*	
			↔ Total airway inflammation	
			↔ Eosinophils	
			↔ Neutrophils	
			↑ Airway eosinophils	
			* Steroid resistant	
LID/HDM BALB/c female	Sensitization: IN challenge: IN	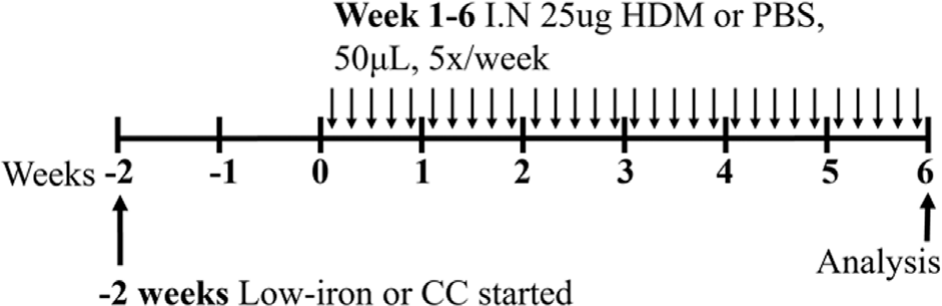	C.f. CC/HDM (normal iron)	Ali et al. (2020a)
			↓ Total airway inflammation	
			↓ Mucus secreting cells	
HID/HDM BALB/c female	Sensitization: IN challenge: IN	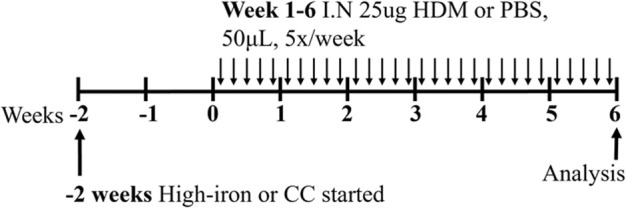	C.f. CC/HDM (normal iron)	Ali et al. (2020a)
			↓ Total airway inflammation	
			↓ Mucus secreting cells	
			↑ Airway eosinophils	

The paucity of knowledge relating to the impact of obesity on respiratory physiology, immune and inflammatory responses, and in the absence or presence of asthma, highlight essential avenues for future research and highlights the importance of refining and interrogating obesity-associated asthma models. Furthermore, it remains to be determined whether reducing adiposity can alter respiratory physiology and AHR, or whether specific dietary nutrients or metabolites can alter respiratory function, and these are important additional avenues for future study.

## 3 Pregnancy and the development and/or severity of asthma in offspring

### 3.1 Effects of low systemic iron status during pregnancy effects on offspring

Increased lung iron status is linked to the pathogenesis and severity of asthma, cystic fibrosis, COPD, and idiopathic pulmonary fibrosis (Reid et al., 2007; Ghio et al., 2013; Cloonan et al., 2017; Ali et al., 2020a; Ali et al., 2020b). Interestingly, lower systemic iron status is also implicated in the development of asthma. Low systemic iron, as measured by exhaled breath condensate, is associated with asthma in both adults and children (Ramakrishnan and Borade, 2010; Vlasic et al., 2011; Brigham et al., 2015). Additionally, lower maternal iron status during pregnancy has been linked with poorer respiratory outcomes in children (Triche et al., 2011; Bedard et al., 2018). Maternal anemia in pregnancy is linked to early-life recurrent wheeze and poorer childhood lung function, as measured by FEV_1_, FVC, and physician-diagnosed asthma at age six (Triche et al., 2011; Bedard et al., 2018). Lower maternal hemoglobin concentration during pregnancy is also linked with elevated IgE and risk of allergic sensitization in children (Shaheen et al., 2017).

Intergenerational models of maternal systemic iron status have predominantly been studied in the context of brain development in offspring (Juul et al., 2019). A recent study modeling diet-induced reduction in systemic iron status during pregnancy found that lower maternal iron status resulted in offspring with impaired lung function at baseline, and increased AHR, airway inflammation, and small airway collagen deposition (Gomez et al., 2021). However, studies modeling altered iron status during pregnancy that examine lung function and structure in offspring are limited.

Genetically-induced changes to iron status provide invaluable enhancements to our understanding of physiological changes from altered iron status and are essential in modeling genetic disorders, such as hereditary hemochromatosis, which is an iron overload disorder that is caused by mutations in the Homeostatic Iron Regulator (HFE) gene. HFE^−/−^ mouse models of iron overload have advanced our understanding of physiological processes involving iron isotype regulation and compositions of numerous organs and cell types (Albalat et al., 2021). A recent study used an iron overload model (*Hamp* knockout) to show that maternal hepcidin, rather than embryonic hepcidin, regulates embryonic iron levels (Sangkhae et al., 2020). Systemic iron deficiency has also been modeled using transthyretin-hepcidin transgenic mice that overexpress hepcidin (Nicolas et al., 2002), and ferroportin ablation in intestinal epithelial cells to eliminate intestinal iron absorption resulting in end-stage iron deficiency anemia (Schwartz et al., 2019).

Genetic disease models are valuable tools for defining mechanisms of pathogenesis via gain or loss of function experiments. However, protocols that involve diet-induced alterations to iron status are essential for comparing physiological processing of various forms of iron. This has been demonstrated by a study that compared ferrous and ferric iron absorption, whereby examination of the effects of diet-induced iron sufficient status (35 mg Fe/kg) and iron deficiency (2 and 20 mg Fe/kg) showed that ferric iron, compared to ferrous iron, may be a more effective iron supplement due to its slow release and reduced rate of absorption (Aslam et al., 2014).

Despite these advances, there remains limited understanding of the mechanisms underpinning the clinical associations between systemic iron deficiency during pregnancy and childhood asthma and allergy, which highlights the importance of utilizing intergenerational iron deficiency and asthma models to examine these associations. In this scenario, diet-induced models of iron deficiency are the most clinically relevant approach and would facilitate assessments of the effects of different iron supplementation formulae during pregnancy on respiratory outcomes in offspring.

### 3.2 Environmental exposures during pregnancy and the development and/or severity of asthma in offspring

The fetal origins of adult disease hypothesis proposes that the *in utero* environment is critical for determining an individual’s susceptibility to certain chronic diseases later in life (Barker, 1990). Notably, suboptimal nutritional status, such as undernutrition due to maternal smoking or maternal exposure to heavily polluted air, as well as toxins inhaled or ingested by pregnant mothers, play key roles in fetal underdevelopment and organ dysfunction after birth (Wang et al., 2020; Chen et al., 2021; Li et al., 2021). The latent and persistent effects of fetal programming may arise from epigenetic modifications that permanently change key regulators of a range of biological processes, including cellular metabolism (e.g., mitochondrial function) and immune responses (e.g., heightened pro-inflammatory mediator production) with or without additional external environmental stimuli after birth (Barker and Osmond, 1986; Petronis, 2010; Li et al., 2018; Wang et al., 2020; Chen et al., 2021).

In humans, lung morphogenesis begins at 3 to 4 weeks post-conception, followed by five stages of intense development (embryonic, pseudoglandular, canalicular, saccular, and alveolar) that occur up to 36 weeks post-conception, and completion of lung development during childhood and young adulthood. *In utero* environmental insults during any of the developmental stages can result in abnormal lung architecture (Li et al., 2021) and function (Harju et al., 2016; Lee et al., 2018), and increased susceptibility to asthma (Hazlehurst et al., 2021) and asthma exacerbations after birth (Gilliland et al., 2001). Tobacco cigarette smoke and particulate matter (PM2.5) are currently the most commonly occurring *in utero* toxins that impair lung function in offspring and increase their susceptibility to asthma (Li et al., 2018; Wang et al., 2021b; Chen et al., 2021).

Epidemiological studies suggest that maternal smoking and inhalation of heavily polluted air are linked to intrauterine underdevelopment, preterm birth, and small birth weight (Wang et al., 2020). As a result, lung development and function are inevitably affected (Hayatbakhsh et al., 2009; Joubert et al., 2016). Notably, low birth weight and impaired lung function are strongly linked with the increased prevalence of childhood wheezing and asthma (Turner et al., 2009; Xu et al., 2014). Both tobacco cigarette smoke and PM2.5 contain thousands of chemical substances, including free radicals, that possess strong oxidative properties. As a result, oxidative stress has been proposed as the common mechanism that translates maternal exposure to cigarette smoke and PM2.5 into inflammatory responses and pathology in the lung in offspring (Sukjamnong et al., 2017; Wang et al., 2021b). Indeed, maternal exposure to tobacco cigarette smoke and combustion-generated PM induced systemic oxidative stress in offspring and greater AHR (Wang et al., 2013; Zacharasiewicz, 2016; Wang et al., 2021b).

Increased pro-inflammatory cytokine responses, including that of IL-8, and T_H_2 hyperactivity reflected by increased IL-5, IL-9, and IL-13 levels, have been detected in the cord blood of newborns from smoking mothers (Noakes et al., 2003; Chahal et al., 2017). However, some infants born to smokers also show changes in toll-like receptor (TLR) 2 function and attenuation of TNF-α, IL-6, and IL-10 levels indicating decreased innate immune responses (Noakes et al., 2003). In animal models, T helper cells including T_H_1, T_H_2, T_H_17, and T regulatory cells were reduced in offspring born to mothers with *in utero* PM exposure (Wang et al., 2013). AHR is also present in the absence of additional ova challenge in adult mice suggesting that this is a prolonged effect of *in utero* exposure (Wang et al., 2021b). Alarmingly, these adverse effects in offspring still occur as a result of maternal inhalation of air pollutants that are present at levels considered safe, and these sequelae are not mitigated by migrating to a pollutant-free environment after conception (Wang et al., 2021b). Thus, the *in utero* effect on AHR most likely involves epigenetic changes and the maternally-derived mitochondrial DNA without modification of genomic profiles (Shrine et al., 2019). A meta-analysis of 13 cohort studies identified more than 3,000 CpG islands that are differentially methylated due to maternal smoking (Shrine et al., 2019). Some of these effects, such as hypermethylation of ESR1 and CpGs in IL-32, have been linked with the pathogenesis of asthma (Shrine et al., 2019). In this scenario, PM exposure can initially cause changes in maternal DNA methylation (Ferrari et al., 2019) that are then inherited by the offspring, which could explain why migrating to a pollutant-free area during pregnancy is not sufficient to mitigate the risk of asthma development in offspring (Wang et al., 2021b). Additionally, epigenetic modifications to the fetal genome during *in utero* PM exposure can further increase the risk of asthma, such as the antioxidant superoxide dismutase 2 (SOD2) promoter DNA methylation, and TMEM184A which is involved in the inflammatory response (Zhou et al., 2019; Parikh et al., 2022). In summary, maternal exposure to inhaled toxins, such as tobacco smoke and PMs, can increase the risk of asthma in offspring via multiple mechanisms, including impaired lung development and structure, oxidative stress, immune response, and epigenetic modifications.

## 4 Conclusions and potential avenues of investigation

Refinements and innovation in technologies to assess lung function in mouse models that recapitulate the key features of asthma and different endo/phenotypes of severe asthma have significantly advanced the understanding of disease-causing mechanisms. This has led to improvements in the clinical management of the disease, however, there remains many endo/phenotypes of severe asthma that have no effective treatment options. The use of highly representative mouse models (Tables 1–3) in combination with diet and environmental exposures, are key to increasing the understanding of mechanisms of pathogenesis, including drivers of AHR, and should include comprehensive immune profiling and remodeling assessments. These models coupled with technological advances in measuring respiratory physiology, will lead to the identification of new therapeutic targets that support the goal of achieving disease remission.
